# Association Between the Mitral Annular E/e′ Ratio and In-Hospital Mortality in Acute ST-Elevation Myocardial Infarction: A Prospective Observational Study

**DOI:** 10.7759/cureus.103805

**Published:** 2026-02-17

**Authors:** Ashish K Jain, Ajay Sharma, Raghvendra S Meena, Bhushan Shah, Aditi Mohta, Sudesh Prajapathi, Shivankur Singh, Akshyaya Pradhan

**Affiliations:** 1 Department of Cardiology, All India Institute of Medical Sciences, Bhopal, IND; 2 Department of Cardiology, Gandhi Medical College, Bhopal, IND; 3 Department of Community Medicine, People's College of Medical Sciences and Research Centre, People's University, Bhopal, IND; 4 Department of Cardiology, King George's Medical University, Lucknow, IND

**Keywords:** acute st-elevation myocardial infarction, diastolic function, e/e' ratio, lv filling pressure, tissue doppler imaging

## Abstract

Background

Left ventricular (LV) diastolic dysfunction and elevated LV filling pressures are associated with adverse outcomes after acute ST-elevation myocardial infarction (STEMI). The tissue Doppler-derived mitral annular E/e′ ratio provides a simple, noninvasive estimate of LV filling pressure; however, its prognostic value when measured early after admission remains unclear. This study evaluated the association between early mitral annular E/e′ ratio and in-hospital mortality in patients with acute STEMI.

Materials and methods

In this prospective observational study, 256 consecutive patients admitted with acute STEMI underwent transthoracic echocardiography with tissue Doppler imaging within 24 hours of admission. Mitral inflow velocities (E, A), mitral annular early diastolic velocity (e’), and E/e’ ratio were measured. Patients were followed until hospital discharge. The primary endpoint of the study was all-cause in-hospital mortality.

Results

Seventy-seven patients (30%) had an elevated E/e’ ratio (>15), suggestive of increased LV filling pressures. In-hospital mortality was significantly higher in patients with E/e’ >15 compared with those with E/e’ ≤15 (34% vs. 4%, p = 0.002). Elevated E/e’ was also associated with reduced LV ejection fraction and abbreviated deceleration time. After multivariable analysis, E/e′ >15 and Killip class ≥II at admission were independently associated with in-hospital mortality.

Conclusions

An elevated mitral annular E/e′ ratio measured within 24 hours of admission is strongly and independently associated with in-hospital mortality in acute STEMI. Together with the Killip class, E/e′ offers complementary prognostic information and may enhance early bedside risk stratification.

## Introduction

Echocardiography has become the most widely used noninvasive modality for assessing cardiac structure and function in clinical practice. Early myocardial injury following acute coronary syndrome initiates a cascade of adverse remodeling driven by inflammation, endothelial dysfunction, and neurohormonal activation. Conventional echocardiographic predictors of poor outcome, such as left ventricular ejection fraction (LVEF) and restrictive filling pattern, have recently been supplemented by tissue Doppler imaging.

Killip classification remains the cornerstone clinical tool for early risk stratification in acute STEMI, providing a rapid bedside assessment of heart failure severity. However, the Killip classification is based on clinical signs that may reflect overt pulmonary congestion and hemodynamic compromise, potentially underestimating early or subclinical elevations in left ventricular (LV) filling pressures. In contrast, tissue Doppler-derived E/e′ provides a quantitative and noninvasive estimate of LV diastolic pressure that may detect hemodynamic abnormalities before they become clinically apparent.

Advanced LV diastolic dysfunction predicts a poorer prognosis after acute myocardial infarction (MI) [1-3]. In particular, echocardiographic indexes of elevated LV filling pressures are associated with adverse remodeling, an increased incidence of heart failure, and worse survival [1,4]. The deceleration time (DT) of early transmitral flow is the most extensively investigated echocardiographic diastolic parameter and appears to be the simplest and most powerful predictor of outcome [5,6].

Although invasive cardiac catheterization is the gold standard for assessing LV filling, it carries a risk of subsequent complications [7]. In this regard, echocardiography using tissue Doppler imaging has emerged as a valuable noninvasive tool, with predictive value comparable to cardiac catheterization [8]. Doppler tissue imaging of the mitral valve annulus appears to be particularly useful, and reduced early mitral annulus velocity (e′) indicates impaired myocardial relaxation [9]. Unlike other Doppler parameters of diastolic function, e′ velocity appears to be relatively independent of preload, especially when the rate of myocardial relaxation is decreased [10].

Additionally, the ratio of early trans-mitral flow velocity (E) to early diastolic septal mitral annulus velocity (E/e′) correlates well with LV diastolic dysfunction [11,12]. The prognostic value of E/e′ >15 following MI has been evaluated previously in a study by Hillis et al. [9], who indicated that E/e′ is a powerful predictor of survival after acute MI. Subsequently, the E/e′ ratio has become central to guidelines for the evaluation of LV diastolic function [13].

Existing evidence supporting the prognostic utility of E/e′ following acute MI is derived largely from Western populations and earlier treatment eras. Given the distinct cardiovascular risk profile and earlier presentation of coronary artery disease among South Asian populations, data validating tissue Doppler-derived indices of LV filling pressure in contemporary Indian patients with ST-elevation myocardial infarction (STEMI) are limited.

The present study was therefore designed to evaluate the prognostic significance of early E/e′ measurement in a contemporary Indian STEMI cohort, with particular emphasis on short-term, in-hospital mortality. The primary objective of this study was to evaluate the association between early mitral annular E/e′ ratio measured within 24 hours of admission and all-cause in-hospital mortality in patients with acute STEMI. Secondary exploratory objectives included assessing the relationship between E/e′ and other echocardiographic indices of LV systolic and diastolic function, as well as its association with baseline clinical and hemodynamic characteristics.

## Materials and methods

Study design and population

This prospective observational cohort study enrolled consecutive patients presenting with acute STEMI at a tertiary care hospital between January 1, 2022, and October 31, 2022. The study was designed to evaluate the association between tissue Doppler-derived indices of LV diastolic function and early hemodynamic status in the acute phase of STEMI. The study protocol was approved by the Institutional Ethics Committee of Gandhi Medical College (approval number: 27358/MC/IEC/2021), and the study was conducted in accordance with the Declaration of Helsinki. Written informed consent was obtained from all participants prior to enrollment.

Inclusion and exclusion criteria

Patients were eligible for inclusion if they were 18 years of age or older, fulfilled European Society of Cardiology/American College of Cardiology diagnostic criteria for acute STEMI, presented within 24 hours of symptom onset, and underwent comprehensive transthoracic echocardiography within 24 hours of hospital admission.

Patients were excluded if they had a prior history of MI or cardiomyopathy, significant primary valvular heart disease other than ischemic mitral regurgitation, atrial fibrillation or other sustained arrhythmias interfering with Doppler analysis, congenital heart disease, prior cardiac surgery, poor acoustic windows precluding adequate image acquisition, hemodynamic instability requiring mechanical circulatory support at the time of echocardiography, or serious noncardiac illness limiting short-term prognosis.

Clinical assessment and management

Baseline demographic data, cardiovascular risk factors, clinical presentation, and laboratory parameters were recorded at admission. Clinical heart failure severity was assessed at presentation using the Killip classification and dichotomized as Killip class I vs ≥II. Infarct location was determined from admission electrocardiography and categorized as anterior or non-anterior STEMI. The infarct-related artery was identified angiographically when available and classified as left anterior descending (LAD) or non-LAD. Reperfusion therapy, including thrombolysis or primary percutaneous coronary intervention, was administered according to contemporary guideline-directed management. Door-to-needle time was recorded for patients undergoing thrombolytic therapy and defined as the interval from hospital arrival to initiation of fibrinolysis. Failed thrombolysis was defined as <50% resolution of ST elevation at 90 minutes after thrombolytic administration or the need for rescue percutaneous coronary intervention. All patients received standard medical therapy unless contraindicated. Patients were followed prospectively from admission until hospital discharge.

Echocardiographic acquisition

Transthoracic echocardiography, including tissue Doppler imaging, was performed within 24 hours of hospital admission and after initiation of reperfusion therapy (thrombolysis or primary percutaneous coronary intervention), reflecting early post-reperfusion LV hemodynamic status. Transthoracic echocardiography was performed using a commercially available ultrasound system (HP Sonos 5500, Philips Medical Systems, Andover, MA, USA) equipped with a 2-4 MHz transducer. All studies were acquired by a single experienced echocardiographer who was blinded to clinical and laboratory data at the time of image acquisition. Intra-observer variability was evaluated in a randomly selected subset of patients and demonstrated acceptable reproducibility for key Doppler and tissue Doppler parameters.

Standard parasternal and apical views were obtained in accordance with American Society of Echocardiography recommendations [13,14]. Measurements were averaged over three consecutive cardiac cycles. LVEF was calculated using the biplane modified Simpson method. LV dimensions were measured using two-dimensional guided M-mode imaging.

Doppler and tissue Doppler analysis

Mitral inflow velocities were assessed using pulsed-wave Doppler in the apical four-chamber view, with a 1-2 mm sample volume positioned at the tips of the mitral valve leaflets and the Doppler beam aligned parallel to transmitral flow. Recordings were obtained during end-expiratory apnea. Peak early diastolic (E) velocity, peak late diastolic (A) velocity, E/A ratio, and E-wave DT were measured. A restrictive filling pattern was defined as an E/A ratio ≥2, or an E/A ratio between 1 and 2 in the presence of an E-wave DT ≤140 ms [15]. Color Doppler imaging was used to assess the severity of mitral regurgitation semi-quantitatively from apical four- and two-chamber views. Mitral regurgitation was graded as mild, moderate, or severe based on the ratio of regurgitant jet area to left atrial area [16].

Tissue Doppler imaging of the mitral annulus was performed from the apical four-chamber view by placing the sample volume sequentially at the septal and lateral annular sites. Early diastolic annular velocities (e′) were measured, and the average of septal and lateral values was used for analysis. The E/e′ ratio was calculated as a noninvasive estimate of LV filling pressures. An E/e′ ratio >15 was prospectively defined as indicative of elevated LV diastolic pressure [15].

Study variables and outcomes

The echocardiographic variables analyzed included LVEF, LV dimensions, mitral inflow E and A velocities, E/A ratio, E-wave DT, mitral annular e′ velocity, and E/e′ ratio. The primary outcome of the study was all-cause in-hospital mortality. The primary exposure variable was the mitral annular E/e′ ratio measured within 24 hours of admission. Secondary analyses were exploratory and evaluated the associations between E/e′ and other echocardiographic parameters of LV structure and function, as well as baseline clinical and hemodynamic characteristics.

Statistical analysis

Sample size was calculated for a two-proportion comparison of in-hospital mortality (30% vs 5%) using E/e′ >15 vs ≤15, with a two-sided α of 0.05 and 80% power, yielding 64 patients per group [9]. Assuming a 30% prevalence of elevated E/e′ and allowing for missing data, the final sample size was set at 256 patients.

Statistical analyses were performed using SPSS Statistics version 17.0 (IBM Corp. Released 2008. IBM SPSS Statistics for Windows, Version 17.0. Armonk, NY: IBM Corp.). Continuous variables were expressed as mean ± standard deviation, and categorical variables as frequencies and percentages. Between-group comparisons were performed using the chi-squared test for categorical variables. Multivariable logistic regression analysis was performed to identify independent predictors of in-hospital mortality. Variables were selected based on clinical relevance and prior literature. Covariates included age, infarct location, LVEF, Killip class at admission, mitral regurgitation severity, reperfusion strategy, troponin I, and the mitral annular E/e′ ratio. The mitral annular E/e′ ratio was entered into the model as a categorical variable, with a predefined cut-off of >15. Variance inflation factors were calculated to assess multicollinearity among covariates prior to multivariable modelling. Adjusted odds ratios with 95% confidence intervals were reported. A two-sided p-value < 0.05 was considered statistically significant.

## Results

Study population and baseline characteristics

Between January 1, 2022, and October 31, 2022, 256 consecutive patients presenting with acute STEMI were enrolled in this prospective observational study. Baseline demographic, clinical, hemodynamic, and laboratory characteristics are summarized in Table 1. The study cohort comprised 210 men (82%) and 46 women (18%), with a mean age of 54.9 ± 11.2 years. When stratified by E/e′ category, patients with E/e′ >15 were significantly older and had a higher prevalence of diabetes mellitus, whereas the prevalence of hypertension and current smoking was similar between groups. Anterior wall STEMI and LAD artery involvement were more frequent among patients with E/e′ >15. In addition to demographic and clinical variables, baseline hemodynamic parameters at admission were analyzed. Patients with an elevated E/e′ ratio (>15) had lower systolic and diastolic blood pressures and higher heart rates than those with E/e′ ≤15, reflecting greater early hemodynamic compromise. Mean systolic blood pressure and diastolic blood pressure were significantly lower in the E/e′ >15 group, while heart rate was significantly higher (all p < 0.01). Troponin I levels were significantly higher among patients with E/e′ >15 compared with those with E/e′ ≤15. Given the skewed distribution of troponin values, results are presented as median (interquartile range). These findings suggest that patients with elevated E/e′ not only exhibited more advanced diastolic dysfunction but also demonstrated greater biochemical and hemodynamic severity at presentation. Clinical heart-failure severity at presentation differed markedly, with Killip class ≥II observed in 75.3% of patients with E/e′ >15, compared with 20.7% in those with E/e′ ≤15 (p < 0.001). Door-to-needle time among thrombolyzed patients was comparable between E/e′ categories. Moderate-to-severe mitral regurgitation was significantly more frequent among patients with E/e′ >15. Additionally, patients with E/e′ >15 had significantly higher rates of failed thrombolysis and required inotropic support more frequently, reflecting greater baseline clinical and hemodynamic severity (Table 1).

**Table 1:** Baseline demographic, clinical, hemodynamic, and laboratory characteristics Values are presented as mean ± SD, median (interquartile range), or n (%). STEMI: ST-elevation myocardial infarction, LAD: left anterior descending, PCI: percutaneous coronary intervention, SD: standard deviation, E/e′: mitral inflow E velocity to annular e′ velocity ratio

Variable	Overall (n = 256)	E/e′ ≤15 (n = 179)	E/e′ >15 (n = 77)	p-value
Age, years (mean ± SD)	54.9 ± 11.2	52.8 ± 10.6	59.6 ± 11.8	<0.001
Male sex, n (%)	210 (82.0)	152 (84.9)	58 (75.3)	0.06
Hypertension, n (%)	64 (25.0)	41 (22.9)	23 (29.9)	0.24
Diabetes mellitus, n (%)	64 (25.0)	37 (20.7)	27 (35.1)	0.01
Current smoker, n (%)	166 (65.0)	118 (65.9)	48 (62.3)	0.59
Anterior wall STEMI, n (%)	179 (70.0)	117 (65.4)	62 (80.5)	0.01
Systolic blood pressure (mmHg), mean ± SD	118 ± 22	122 ± 20	108 ± 23	<0.001
Diastolic blood pressure (mmHg), mean ± SD	74 ± 13	77 ± 12	68 ± 14	0.002
Heart rate (beats/min), mean ± SD	86 ± 16	82 ± 14	95 ± 18	<0.001
Troponin I (ng/mL), median (IQR)	18.4 (9.2-36.7)	15.6 (8.1-29.4)	28.9 (14.5-52.1)	0.001
Infarct-related artery				
LAD, n (%)	170 (66.4)	107 (59.8)	63 (81.8)	<0.001
Non-LAD, n (%)	86 (33.6)	72 (40.2)	14 (18.2)	
Killip class at admission				
Killip I, n (%)	161 (62.9)	142 (79.3)	19 (24.7)	<0.001
Killip ≥II, n (%)	95 (37.1)	37 (20.7)	58 (75.3)	
Reperfusion strategy				
Thrombolysis, n (%)	156 (61.0)	116 (64.8)	40 (51.9)	0.05
Primary PCI, n (%)	100 (39.0)	63 (35.2)	37 (48.1)	
Failed thrombolysis, n (%)	34 (13.3)	15 (8.4)	19 (24.7)	<0.001
Door-to-needle time, min (mean ± SD)	45 ± 20	44 ± 18	47 ± 22	0.48
Inotropic support, n (%)	68 (26.6)	27 (15.1)	41 (53.2)	<0.001
Mitral regurgitation severity				
None/mild, n (%)	205 (80.1)	160 (89.4)	45 (58.4)	<0.001
Moderate–severe, n (%)	51 (19.9)	19 (10.6)	32 (41.6)	

Echocardiographic and Doppler characteristics

The distribution of echocardiographic parameters is presented in Table 2. Mean LV end-diastolic dimension was 44.8 ± 7.4 mm, and mean LV end-systolic dimension was 34.2 ± 7.2 mm. Mean mitral inflow E and A wave velocities were 0.73 ± 0.19 m/s and 0.63 ± 0.15 m/s, respectively. Mean mitral annular e′ velocity was 8.2 ± 2.2 cm/s. The mean E/e′ ratio for the overall cohort was 9.3 ± 3.8. When stratified by infarct location, patients with anterior wall STEMI had lower LVEF and higher E/e′ values than those with non-anterior infarction. Technically adequate measurements of LVEF, mitral inflow Doppler parameters, tissue Doppler-derived mitral annular velocities, and E/e′ ratio were obtained in all patients included in the final analysis. Patients with inadequate acoustic windows or uninterpretable Doppler signals were excluded at enrollment.

**Table 2:** Echocardiographic and Doppler parameters Echocardiographic and Doppler measurements were performed in accordance with the American Society of Echocardiography and the European Association of Cardiovascular Imaging recommendations [14,15]. LV: left ventricle, LVEF: left ventricular ejection fraction, E: peak early diastolic transmitral flow velocity, A: peak late diastolic transmitral flow velocity, E/A ratio: ratio of early to late diastolic transmitral flow velocities, e′: early diastolic mitral annular tissue Doppler velocity, E/e′: ratio of early transmitral flow velocity to early diastolic mitral annular velocity, SD: standard deviation, DT: deceleration time

Parameter	Mean ± SD
LV end-diastolic dimension (mm)	44.8 ± 7.4
LV end-systolic dimension (mm)	34.2 ± 7.2
LVEF (%)	47.1 ± 8.0
Mitral E velocity (m/s)	0.73 ± 0.19
Mitral A velocity (m/s)	0.63 ± 0.15
E/A ratio	1.15 ± 0.36
E-wave DT (ms)	142.6 ± 42.5
Mitral annular e′ (cm/s)	8.2 ± 2.2
E/e′ ratio	9.3 ± 3.8

Distribution of E/e′ ratio and diastolic filling parameters

The distribution of E/e′ ratios in the study population and the cross-tabulation between E/e′ categories and DT are shown in Table 3. An elevated E/e′ ratio (>15), indicative of increased LV filling pressures, was observed in 77 patients (30.1%), while the remaining 179 patients (69.9%) had E/e′ ≤15. Patients with elevated E/e′ demonstrated a higher prevalence of abnormal diastolic filling patterns. Patients with E/e′ >15 more frequently exhibited shortened mitral E-wave DT (<140 ms) compared with those with E/e′ ≤15 (59 (77%) vs 23 (13%), respectively; p < 0.001). DT was analyzed as a categorical variable using a prespecified cutoff of <140 ms, consistent with restrictive filling physiology.

**Table 3:** Association between mitral annular E/e′ ratio and mitral E-wave DT Definitions of mitral inflow DT and E/e′ ratio were based on guideline-recommended criteria for the assessment of LV diastolic function [13,15]. Between-group comparisons were performed using the chi-squared test (degree of freedom = 1). A p-value <0.05 was considered statistically significant. E/e′: mitral inflow E velocity to annular e′ velocity ratio, LV: left ventricular, DT: deceleration time

DT	E/e′ ≤15 (n = 179)	E/e′ >15 (n = 77)	χ^2 ^statistic	p-value
≥140 ms	156 (87)	18 (23)	97.67	<0.001*
<140 ms	23 (13)	59 (77)		

Unadjusted in-hospital mortality differed markedly across E/e′ categories, with significantly higher mortality observed among patients with E/e′ >15 compared with those with E/e′ ≤15 (34% vs 4%, p < 0.001). A stepwise increase in mortality risk was observed with increasing E/e′ values (p for trend <0.001). In addition to E/e′ and DT, other diastolic indices, including E/A ratio and mitral annular e′ velocity, were evaluated but are not shown in detail due to collinearity with E/e′ and lack of incremental prognostic value.

Relationship between E/e′ ratio and LV systolic function

Reduced LVEF (<45%) was present in 123 patients. Among these, 70 patients (57%) had an E/e′ ratio >15. In contrast, only 7 of 133 patients (5%) with preserved LVEF (≥45%) demonstrated an E/e′ ratio >15 (Table 4).

**Table 4:** Relationship between E/e′ ratio and LVEF LVEF and E/e′ ratio were assessed according to American Society of Echocardiography recommendations [15,16]. Values are presented as n. Between-group comparisons were performed using the chi-squared test (degree of freedom equal to 1). A p-value < 0.05 was considered statistically significant. E/e′: mitral inflow E velocity to annular e′ velocity ratio, LVEF: left ventricular ejection fraction

LVEF category	E/e′ ≤15	E/e′ >15	χ^2 ^statistic	p-value
<45%	53	70	78.61	<0.001
≥45%	126	7		

In addition to categorical analyses, E/e′ was also evaluated as a continuous variable. A significant inverse correlation was observed between E/e′ and LVEF (r = -0.60, p < 0.001), indicating progressively lower systolic function with increasing E/e′ values. Visual inspection of scatter plots confirmed an approximately linear relationship, with no evidence of influential outliers. Despite this association, collinearity diagnostics confirmed acceptable variance inflation factors (<2.5), and both variables were retained for downstream multivariable analyses.

In-hospital mortality

In-hospital mortality occurred in 34 of 256 patients (13.3%). All in-hospital deaths were cardiovascular in origin. The most frequent causes were cardiogenic shock, malignant ventricular arrhythmias, and progressive heart failure. Transthoracic echocardiography was performed within 24 hours of admission in all patients, and all deaths occurred after echocardiographic assessment. The median time from echocardiography to death was three days (interquartile range, 2-5 days). Mortality was significantly higher among patients with E/e′ >15 compared with those with E/e′ ≤15 (26/77 (34%) vs 8/179 (4%), p < 0.001). Patients who died during hospitalization had higher mean E/e′ values than survivors (14.9 ± 3.4 vs 10.6 ± 3.9; p < 0.001) (Table 5). The absolute risk difference was 30%. The unadjusted odds ratio for in-hospital mortality associated with E/e′ >15 was 12.53 (95% CI, 5.14-30.54).

**Table 5:** In-hospital mortality stratified by E/e′ ratio E/e′ ratio cutoff (>15) reflects elevated LV filling pressure as per echocardiographic guidelines [16]. Values are presented as n (%). Between-group comparisons were performed using the chi-squared test (degree of freedom equal to 1). A p-value < 0.05 was considered statistically significant. E/e′: mitral inflow E velocity to annular e′ velocity ratio, OR: odds ratio, CI: confidence interval, LV: left ventricular

E/e′ category	Deaths, n (%)	Survivors, n (%)	χ^2 ^statistic	p-value	Unadjusted OR (95% CI)
≤15 (n = 179)	8 (4)	171 (96)	37.62	<0.001*	Reference
>15 (n = 77)	26 (34)	51 (66)			12.53 (5.14-30.54)

Multivariable analysis

Multivariable logistic regression analysis was performed to identify independent predictors of in-hospital mortality. Variables entered into the model included age, infarct location, LVEF, Killip class at admission, mitral regurgitation severity, reperfusion strategy, troponin I, and the mitral annular E/e′ ratio. After adjustment for these covariates, an elevated E/e′ ratio (>15) remained independently associated with increased in-hospital mortality (adjusted odds ratio, 4.32; 95% confidence interval, 1.71-10.92; p = 0.002). Killip class ≥II at presentation was also independently associated with in-hospital mortality. In contrast, reduced LVEF (<45%), anterior wall MI, age, mitral regurgitation severity, and reperfusion strategy were not independently associated with mortality after multivariable adjustment (Table 6).

**Table 6:** Multivariable logistic regression for in-hospital mortality Echocardiographic variables were defined and measured in accordance with current guideline recommendations [15,16]. E/e′: mitral inflow E velocity to annular e′ velocity ratio, LVEF: left ventricular ejection fraction, STEMI: ST-elevation myocardial infarction, OR: odds ratio, PCI: percutaneous coronary intervention, CI: confidence interval

Variable	Adjusted OR	95% CI	p-value
E/e′ >15	4.32	1.71-10.92	0.002
Killip class ≥II at admission	5.88	2.31-14.97	<0.001
Anterior STEMI	1.21	0.50-2.92	0.67
LVEF <45%	1.84	0.62-5.42	0.27
Moderate-severe mitral regurgitation	2.12	0.80-6.02	0.12
Reperfusion strategy (primary PCI vs thrombolysis)	0.72	0.30-1.64	0.34
Troponin I (per log-unit increase)	1.42	0.95-2.13	0.09
Age (per year increase)	1.02	0.97-1.07	0.41

## Discussion

Echocardiography is an integral component in the evaluation and management of patients with acute MI, providing essential information regarding infarct location, extent, and ventricular function [17,18]. This study provides validation of the prognostic value of E/e′ in an Indian STEMI population that is underrepresented in prior echocardiographic outcome studies. Despite potential ethnic and regional differences in cardiovascular risk burden and disease phenotype, E/e′ demonstrated robust and independent prognostic value, supporting its use as an early risk-stratification tool across diverse patient populations. In the present prospective observational study, we assessed the prognostic value of echocardiographic parameters, with particular emphasis on tissue Doppler-derived indices of LV diastolic function, in patients presenting with acute STEMI. In our study, when clinical heart failure severity at presentation was accounted for by inclusion of Killip class in the multivariable analysis, both Killip class ≥II and E/e′ >15 emerged as independent predictors of in-hospital mortality. This finding indicates that the prognostic value of E/e′ is not solely attributable to overt clinical heart failure but reflects complementary hemodynamic information captured early after STEMI.

LV diastolic dysfunction is a common pathophysiological consequence of acute MI, reflecting impaired myocardial relaxation, increased ventricular stiffness, and elevated filling pressures [17]. The E/e′ ratio has been extensively validated as a noninvasive surrogate for LV filling pressure and has demonstrated good correlation with invasively measured hemodynamic parameters [11]. Killip class reflects clinically apparent pulmonary congestion and shock, whereas E/e′ provides a quantitative estimate of LV filling pressure and diastolic stiffness; the persistence of both variables as independent predictors supports the concept that these measures capture related but non-identical dimensions of early hemodynamic risk. Patients with elevated E/e′ also demonstrated greater baseline hemodynamic compromise and higher admission troponin levels, consistent with more extensive myocardial injury and impaired ventricular performance. Lower blood pressures and higher heart rates in this subgroup likely reflect early hemodynamic stress and neurohormonal activation following acute infarction. Despite these associations, E/e′ remained independently predictive of in-hospital mortality after multivariable adjustment, suggesting that it captures a distinct and clinically relevant dimension of early filling pressure-related risk beyond infarct size or overt heart failure alone.

Our findings are consistent with those of Hillis et al., who demonstrated that E/e′ was a powerful predictor of survival after acute MI in an unselected cohort followed for a median duration of 13 months [9]. Importantly, the prognostic value of E/e′ in that study was incremental to conventional echocardiographic measures of systolic and diastolic function and established clinical variables. The present study extends these observations by demonstrating that E/e′ also provides independent prognostic information for short-term, in-hospital mortality in patients with acute STEMI. Similar observations regarding the prognostic utility of echocardiographic indices after the first acute MI have been reported by Obeidat et al. [19].

In the current study, impaired LV systolic function was more frequently observed among patients with elevated E/e′ ratios. Although reduced LVEF and anterior wall MI were associated with adverse outcomes on univariable analysis, these variables did not retain independent prognostic significance after adjustment for age and other clinical factors. In contrast, an elevated E/e′ ratio (>15) remained independently associated with in-hospital mortality on multivariable analysis, indicating that E/e′ provides incremental prognostic information beyond conventional systolic and clinical predictors in the early post-reperfusion phase of STEMI [20,21].

Our findings should also be interpreted in the context of contemporary STEMI cohorts treated predominantly with primary percutaneous coronary intervention. In such PCI-era cohorts, early mortality is generally lower, and outcomes are increasingly influenced by rapid restoration of coronary flow, microvascular dysfunction, and early reperfusion injury rather than infarct size alone. Differences in reperfusion strategy, patient profiles, and the timing of echocardiographic assessment may therefore contribute to variability across studies in the relative prognostic importance of systolic versus diastolic indices, particularly in the early phase following STEMI.

From a clinical perspective, an elevated E/e′ ratio should be interpreted as a marker of increased early risk rather than as a stand-alone trigger for specific therapeutic interventions. Its principal value lies in identifying patients with elevated LV filling pressures who may benefit from closer clinical monitoring, cautious volume management, and heightened vigilance for the development of heart failure during hospitalization. Incorporation of E/e′ into early echocardiographic assessment may therefore complement established clinical risk stratification tools, facilitating more individualized care in the acute STEMI setting.

Despite its robustness, the E/e′ ratio has recognized limitations and should be interpreted in the context of the overall clinical and echocardiographic assessment. Conditions such as severe mitral annular calcification, significant mitral stenosis, moderate-to-severe mitral regurgitation, advanced LV systolic dysfunction, and hypertrophic cardiomyopathy may affect the accuracy of E/e′ as an estimate of filling pressure [7]. These considerations are particularly relevant in the setting of acute MI and underscore the importance of comprehensive echocardiographic evaluation.

The lack of independent prognostic significance of LVEF in the fully adjusted model warrants careful interpretation. LVEF and E/e′ capture overlapping aspects of ventricular dysfunction, and their moderate correlation suggests partial redundancy in prognostic information. In the acute STEMI setting, E/e′ may more directly reflect early hemodynamic congestion and elevated filling pressures, which are closely linked to short-term, in-hospital outcomes. When correlated echocardiographic variables are included simultaneously in multivariable models, the number of observed events relative to the number of predictors may reduce the precision of individual effect estimates, resulting in attenuation of statistical significance for one variable without negating its established physiological and prognostic relevance. This study benefits from prospective, consecutive enrollment, early standardized echocardiographic assessment, and blinded image acquisition, all of which enhance methodological rigor and internal validity.

Several limitations of the present study merit consideration. This was a single-center observational study, which may limit the generalizability of the findings. The echocardiographic measurements were obtained at a single time point early in the hospital course, and temporal changes in LV filling pressures during hospitalization were not assessed. Potential confounding factors, such as adherence to medical therapy, were not included in the analysis. The follow-up was limited to the in-hospital period, precluding assessment of long-term outcomes. Finally, the event-per-variable ratio approaches the threshold for multivariable model overfitting, as indicated by relatively wide confidence intervals. Larger studies with higher event counts and longer follow-up durations are warranted to obtain more precise estimates and to further clarify the prognostic role of E/e′ in patients with acute MI.

## Conclusions

In this prospective observational study of patients with acute STEMI, an elevated mitral annular E/e′ ratio (>15) measured within 24 hours of hospital admission was associated with increased in-hospital mortality. These findings should be interpreted as associative rather than causal. The E/e′ ratio may be viewed as a complementary bedside echocardiographic marker that provides incremental information beyond established clinical and systolic parameters, rather than as a standalone risk-stratification tool. While early assessment of E/e′ may help identify patients at higher risk who warrant closer monitoring during hospitalization, external validation in larger, multicenter cohorts is required before routine incorporation into guideline-directed risk stratification pathways can be recommended.
